# Large data and Bayesian modeling—aging curves of NBA players

**DOI:** 10.3758/s13428-018-1183-8

**Published:** 2019-01-25

**Authors:** Nemanja Vaci, Dijana Cocić, Bartosz Gula, Merim Bilalić

**Affiliations:** 1 Department of Psychiatry, University of Oxford, Oxford, UK; 2 Department of Psychology, University of Northumbria at Newcastle, Tyne, UK; 3 Institute of Psychology, University of Klagenfurt, Klagenfurt, Austria

**Keywords:** Bayesian modeling, Big data, Aging, Motor expertise, Skill development, Lifespan psychology, Basketball

## Abstract

Researchers interested in changes that occur as people age are faced with a number of methodological problems, starting with the immense time scale they are trying to capture, which renders laboratory experiments useless and longitudinal studies rather rare. Fortunately, some people take part in particular activities and pastimes throughout their lives, and often these activities are systematically recorded. In this study, we use the wealth of data collected by the National Basketball Association to describe the aging curves of elite basketball players. We have developed a new approach rooted in the Bayesian tradition in order to understand the factors behind the development and deterioration of a complex motor skill. The new model uses Bayesian structural modeling to extract two latent factors, those of development and aging. The interaction of these factors provides insight into the rates of development and deterioration of skill over the course of a player’s life. We show, for example, that elite athletes have different levels of decline in the later stages of their career, which is dependent on their skill acquisition phase. The model goes beyond description of the aging function, in that it can accommodate the aging curves of subgroups (e.g., different positions played in the game), as well as other relevant factors (e.g., the number of minutes on court per game) that might play a role in skill changes. The flexibility and general nature of the new model make it a perfect candidate for use across different domains in lifespan psychology.

Describing the changes in motor and cognitive skills over the human lifespan is an important topic in psychology. For example, developmental psychology is interested in continuous changes in cognitive and physical domains with age (Grusec, 1992), research into aging looks at how older adults deal with the unavoidable decline of general and domain-specific abilities (Salthouse, 2004), whereas expertise researchers are interested in the development and retention of a particular skill over the course of a person’s life (Vaci, Gula, & Bilalić, 2015). The investigation of changes affecting complex skills over the course of the human lifespan poses several problems. The time scale of such changes, as well as the complexity of the skills in question, render laboratory experiments impracticable. Similarly, longitudinal studies are difficult to conduct and are consequently rather scarce. Most studies rely on cross-sectional data that suffer from a number of problems (Hedden & Gabrieli, 2004). Here, we exploit the existence of historical records of a complex motor skill, basketball play, to demonstrate changes in the skill levels of elite athletes. We analyze a sample of over five decades’ worth of data from the National Basketball Association (NBA) using a new way of dealing with such data that is based on Bayesian structural modeling. We extract latent factors of development and aging and show how they differ depending on other external factors, such as the particular position occupied by a player during the game or a player’s activity level (e.g., number of minutes).

## Age-related changes

Age-related changes are commonly assumed to bring a consistent decrease over the course of the human lifetime. Physiological and biological indicators such as muscle strength, endurance, contraction time, and the number of fibers in a muscle all increase throughout childhood, reaching their peak in early adulthood around the age of 25. The decrease is initially slow, until about the age of 50, after which there is a rapid decrease in basic motor indicators (Booth, Weeden, & Tseng, 1994; Faulkner, Larkin, Claflin, & Brooks, 2007; Goodpaster et al., 2006; Rogers & Evans, 1993; Thelen, 2003). Similarly, general cognitive abilities such as processing speed and working memory decline as otherwise healthy adults age (Salthouse, 2010, 2016; Verhaeghen & Salthouse, 1997). Just as with the motor indicators, negative age-related changes in general cognitive abilities start in people’s 20s or 30s, and are continuous and qualitatively similar throughout adulthood (Salthouse, 2016; Verhaeghen & Salthouse, 1997).

On the other hand, there are processes that offset the natural declines of general abilities. They include processes that depend on exercise and accumulated knowledge. The size of the vocabulary is a good example of such a process; another is the motoric skill of rolling cigars, for which knowledge and skill logarithmically increase with experience and age (Crossman, 1959; Keuleers, Stevens, Mandera, & Brysbaert, 2015; McCabe, Roediger, McDaniel, Balota, & Hambrick, 2010). The knowledge of language is a good example of a domain that never decreases over time, in that people always learn new words, but rarely forget the previously learned ones (see Ramscar, Hendrix, Shaoul, Milin, & Baayen, 2014). The power-law increase in the case of vocabulary size is illustrated using a big-data approach by Keuleers and colleagues (2015). Similarly, the declines in the case of chess performance, which strongly depends on knowledge, are much shallower than declines in games or sports that rely on speed of processing (see Vaci et al., 2015). However, motor skills, even basic ones, can also be influenced by the systematic implementation of different types of exercises (Buford et al., 2010; Faulkner et al., 2007; Rogers & Evans, 1993). The question, then, is what happens with complex real-life motor and cognitive skills. These skills inevitably rely on general abilities, which undergo normative age-related decline. However, they also depend on experience and acquired knowledge, for which little or no decline should be expected with age (Salthouse & Maurer, 1996). In other words, even though people experience a decline in the general abilities underpinning their skills, the accumulated knowledge should preserve their skill and slow down the actual decline.

Currently, there is a lack of evidence concerning basic age-related function in real-life skills. Often, researchers do not have the data to model performance measures as people age. In those rare cases in which they have some data, there are usually not enough data points to capture the intricacy of the nonlinear behavior of the age-related function. Here we present a way of using archival data to model the age-related changes in a complex real-life motor skill. We use professional basketball player performance to model age-related changes. The domains of competitive game performance are ideal examples of tasks that depend on both general and domain-specific abilities, where motor speed and power, as well as experience and knowledge, come together to define the level of performance (Bilalić, 2017; Starkes & Ericsson, 2003). Competitive games and sports provide an excellent opportunity for researchers to utilize a well-defined measure of performance and to investigate age-related changes in greater depth (Roring & Charness, 2007; Starkes & Ericsson, 2003; Vaci & Bilalić, 2017).

Here, we will first illustrate different ways in which practitioners have investigated the basic form of the age-related function in the case of real-life performance, and comment on the potential shortcomings of this common approach to dealing with big data. We proceed by presenting a new way of dealing with the data, which is based on Bayesian latent variable modeling (Vandekerckhove, 2014). Finally, we demonstrate how researchers can use the newly developed Bayesian model, which we call *B-Ianus*. The first letter of the model’s name denotes the Bayesian analytical philosophy that governs the model estimation and its use, whereas “Ianus” refers to the Roman god of beginnings, transitions, duality, passages, and endings. This god is often depicted with two faces, one looking to the past, and another looking to the future. In a way similar to its divine counterpart, our model does the same thing. By modeling two phases of age-related changes, development and aging, we investigate the interactions between them. In other words, once we reach the peak of performance we are asking the question of whether we can predict the future decline (aging) of our performance by knowing the preceding increase to the peak (development).

## Modeling age-related changes

The main goal in lifespan psychology is to examine the general principles of development throughout the human lifespan, that is, to describe the form of age-related changes. Lifespan researchers have three goals: (1) generating knowledge of the interindividual shape of the age function, (2) investigating whether overall function differs between groups and individuals, and (3) understanding how more basic processes, the building blocks of age-related changes, influence these changes (Baltes, 1987; Baltes & Baltes, 1990; Baltes, Reese, & Nesselroade, 1977; Baltes, Staudinger, & Lindenberger, 1999; Lerner, 1984). One of the dominant views in lifespan psychology is the theory of gain–loss relation (Baltes, 1987). This theory states that development at all points in life is a joint expression of features of growth (gain) and decline (loss). In other words, the developmental progression across the lifetime always displays adaptive properties, as well as declining ones. The relation between the gains and losses changes systematically over the lifetime. Childhood is characterized by allocation of resources toward gains, where most of the increases in performance are expected to occur. The middle life period tends to be focused on maintaining a stable level of gains and losses, whereas in old age, resources are directed toward the management of loss. When observing real-life skill performance, the continuous interaction between the two overarching forces of gains and losses that underlie this particular skill often produces a nonlinear function over the years. In the following paragraphs, we provide an overview of modeling approaches that are frequently used to investigate these age-related changes, and we also indicate their limitations when analyzing nonlinear behavior of data.

To capture the nonlinear changes that occur during life, researchers often use polynomial regression, in which age is transformed by the power functions (usually via quadratic transformations). Polynomial regressions result in a nonlinear fit of the relationship between age and performance, which sheds more light on the form of the age-related changes (Goal 1 in lifespan psychology). In lifespan psychology, a second-order polynomial or quadratic function is often the relation of choice when modeling age-dependent changes. These studies indicate that performance follows two phases of development with one transition point. For example, practitioners of speed-dependent sports, such as baseball, peak at the age of about 27 years on most measures of performance, and then start to decline relatively quickly (Allen & Hopkins, 2015; Bradbury, 2009; Brander, Egan, & Yeung, 2014; Dendir, 2016; Hollings, Hopkins, & Hume, 2014; Lailvaux, Wilson, & Kasumovic, 2014; Schulz, Musa, Staszewski, & Siegler, 1994). Similarly, studies investigating cognitive skills such as chess have shown that players improve quickly and reach the peak of performance in their late 30s (Roring & Charness, 2007; Vaci & Bilalić, 2017; Vaci, Gula, & Bilalić, 2014, 2015). After this peak, chess performance starts to decline as people age.

We have recently demonstrated that a third-order polynomial, or cubic function, fits the data better in cognitive domains (Vaci et al., 2015). The cubic function adds a third phase of development in addition to development and decline. The peak performance is indeed followed by a decline, but the decline is not constant. The cubic function reveals a potential third phase of age-related development, in which players stabilize in their performance and preserve it in the face of increased age (Vaci et al., 2015). In comparison to the quadratic relationship, the cubic function adds theoretically relevant implications concerning additional aging stages. This scenario could also represent a significant theoretical and practical difference between the general processes (e.g., memory and reasoning) and domain-specific abilities (e.g., decision making in chess) in their changes across the lifetime.

However, both quadratic and cubic polynomial functions suffer from multiple drawbacks when they are used for analyzing real-life performance. One problem is that individual polynomial coefficients are highly correlated. In situations in which researchers are interested in the underlying factors that influence age-related changes (Goal 3 above), not just the basic form of the function, but also the interaction between variables of interest with polynomial coefficients, can result in strong overfitting of the data. In addition to this problem, the polynomial functions are symmetric around the point of inflections (maximal and minimal value of the function). When applying them to behavioral and psychological data, for example in sports, a sharper decrease of the function after the peak of performance does not necessarily mean a sharper decline. It might simply indicate that this player increased much more quickly and dramatically than other people in the dataset. In other words, the subgroup analysis, one of the main interests of lifespan psychology (Goal 2 above), is problematic. The cubic function alleviates this problem to some extent by adding a third phase and a prolonged tail of the function, which inflects the decrease and indicates that this player is not experiencing a dramatic decline. In other words, it adds more flexibility and degrees of freedom in the model, which can subsequently adapt better to the data. However, every polynomial regression suffers from this problem to the same extent.

One way around the problems inherent in polynomial regression is to use exponential functions. Just like the polynomial function, exponential functions provide us with an understanding of the basic form of changes over the course of life. However, they are more flexible than the polynomial function when fitting sudden changes of performance around the peak value. For example, instead of using a single function, Schroots (2012) utilized double logarithmic functions to describe age-related changes in the functional capacity of people. The first function describes the development before the peak, whereas the second function fits the decrease after the peak. Similarly, Simonton (1997, 2015) used a double-exponential function to describe the creative potential and changes in the output of ideas throughout a person’s life.

Both models are preferable to the common polynomial models, because they adjust the age-related function for the group and individuals much more accurately. In other words, the exponential function allows us to test and compare the behavior of cognitive processes between the groups, as well as between the processes themselves. For example, the model of career trajectories and landmarks showed different rates in the creative potential of practitioners in different fields, indicating that novelists generate original ideas more slowly, and take longer to develop them, than poets do (see Simonton, 1989). These models were also used to illustrate the differences between the lifespan changes in fluid and crystallized abilities (Schroots, 2012). Fluid intelligence follows a two-step function with one inflection point: that is, people increase in fluid abilities until their mid-20s, when they reach the peak, which is followed by constant decline as people age. In the case of crystallized abilities, lifelong changes progress through three stages with two inflection points. People slowly build up crystallized abilities until their late 30s, when they reach the peak. This is followed by maintenance of the abilities, which stay at the same level until old age, when people’s abilities slowly start to decline.

However, these models still have a problem with taking into account the building blocks of age-related changes (Goal 3)—that is, the different factors that influence lifelong function. Additional variables cannot be interacted with exponential coefficients during the model fitting. A possible way around this problem—namely a two-step analysis, in which exponential coefficients are estimated in the first step and regressed jointly with the factors of interest in the second step—suffers from *generated regressor bias* (Pagan, 1984). In other words, using generated values from regression to draw valid inferences in the second step can be problematic, since we are using aggregated results that are sensitive even to small biases in the data. When the resulting value from the first regression suffers from sampling bias, the regression analysis in the second step results in biased estimates and inflated effect sizes and test statistics (Boehm, Marsman, Matzke, & Wagenmakers, 2018).

Finally, the researchers can use nonlinear data-driven methods such as generalized additive models (GAM; see Wood, 2006). The GAM is a data-driven method designed to estimate the nonlinear relation between the covariates and the dependent variable. A generalized additive model is nothing other than a generalized linear model with a linear predictor over a sum of smooth functions of covariates. Therefore, the main goal of this analysis is to estimate the space of functions that can represent the nonlinear shape of the data (see van Rij, Vaci, Wurm, & Feldman, 2018). In contrast to the standard linear model, in GAM we do not have to specify the function (polynomial terms, exponential equation, etc.), because it iteratively optimizes the smooth function (basis) and proposes an optimal structure between the dependent and independent variables. The main problem with the nonlinear methods is that the results of the GAM model cannot be interpreted in standard linear regression terminology, in which the results tell us about the change in the dependent variable for one unit increase or decrease in the independent variable. The GAM provides information about the wiggliness of the regression line (summarization of all individual functions), and whether the function is significantly different from zero. As in the case of most data-driven and nonlinear methods, the visualization is a necessary tool when interpreting the results, whereas we cannot quantify the information behind the estimated parameters.

Here we primarily used exponential functions to model the changes with age seen in expert performance in basketball. We modeled the age-related changes separately before and after the peak. We do, however, also provide additional analysis in which we calculated the inflection point, the exact age when the increase in performance turns negative, using the Heaviside functions (Bracewell, 1986). In addition to only modeling the age-related changes, we propose a structural model constructed using a Bayesian latent cognitive variable modeling approach, which offers more information on the research questions proposed in the domain of lifespan psychology. We show how this model can be used on natural and large datasets to investigate age-related changes, as well as the different factors that influence these changes. Finally, we quantify the relationship between two factors and show how development (the prepeak increase) interacts with aging (postpeak decline).

## Method

### Dataset and measures of basketball skill

Unlike many real-life domains, in competitive games and sports it is possible to quantify the skill of players using performance measures (Franks & Goodman, 1986). In the case of this study, we used large datasets that measure basketball performance (“Basketball Statistics and History,” n.d.). This type of dataset, which collects demographic and performance-level variables for players who compete at a professional level, is usually maintained by official sport federations. Since there are multiple ways of quantifying basketball performance, we chose the three measures that are most commonly used in today’s basketball performance analyses: win shares (WS), value over replacement player (VORP), and player efficiency rating (PER; for more information, see Kubatko, Oliver, Pelton, & Rosenbaum, 2007). WS is an estimate of players’ contributions (to the team) in terms of wins: it attempts to allot shares in the credit for a team’s success to the individuals on that team (Kubatko, n.d.; Oliver, 2004). WS is widely used, since it is one of the only measures that takes into account both the defensive and offensive contributions of players to a team’s win (while other measures rely more on offense-related statistics). The WS measure also takes into account the time the player spent on court as well as the pace of the game during the player’s time on court. VORP is an estimate of each player’s overall contribution to the team, measured against what a theoretical replacement would provide (the replacement being either a player given a minimum salary or a player who is not a regular part of the team’s rotation: Myers, n.d.; see also Barzilai & Ilardi, 2008). It is an estimate of the number of points a player is producing above or below a replacement player per 100 team possessions in a season. Even though it is standardized for a specific season (thus allowing players that played during the same season to be compared), VORP relies heavily on offense-related statistics, and hence is not a very good defense measurement tool. PER is a measure of how productive and efficient a player is during the time spent on court (“Calculating PER,” n.d.; Hollinger, 2002, 2011).

It is clear that all three measures have their own advantages and disadvantages. Given that the topic of this article is modeling of the aging function of motor expertise, we have chosen to showcase, in the main text, the analysis conducted on the WS measure, because it is the only one of the three that takes into account a broader range of abilities (e.g., defense and offense). However, since WS, unlike the other two measures, is lacking in standardization and does not take into account the pace of the game and the time spent of court, we also conducted analyses on PER and VORP, and these can be found in the supplemental materials and the Appendix.

We illustrate our B-Ianus model on WS in the main text, and provide the same analyses for the other two measures in the Appendix. The details and descriptive statistics on the complete data, including cross-validation on the polynomial regressions and all following investigations, are described in the online materials, together with accompanying R codes (https://osf.io/yhmja/). We structured our analyses around the three goals of lifespan psychology: (1) describing the changes in performance over the course of the careers of NBA players; (2) the different career trajectories of different groups, in this case the position they play in the game; and (3) investigating how other factors, in this case the playing time, influence the age-related function. The first analysis, on the form of age-related changes, we conducted using the whole sample, which contains 50 years’ and 2,845 players’ worth of data. The second two analyses, on the subgroups and activity (minutes per game played), were performed on a randomly chosen sample of 400 basketball players.^1^ All analyses are carried out as a methodological illustration of the model, which can be employed in different domains. In this particular case, player position and time spent on court were among the available variables. In other domains, other variables may be available that are more pertinent than those used here.

### Goal 1: The form of age-related changes

**Fig. 1:**
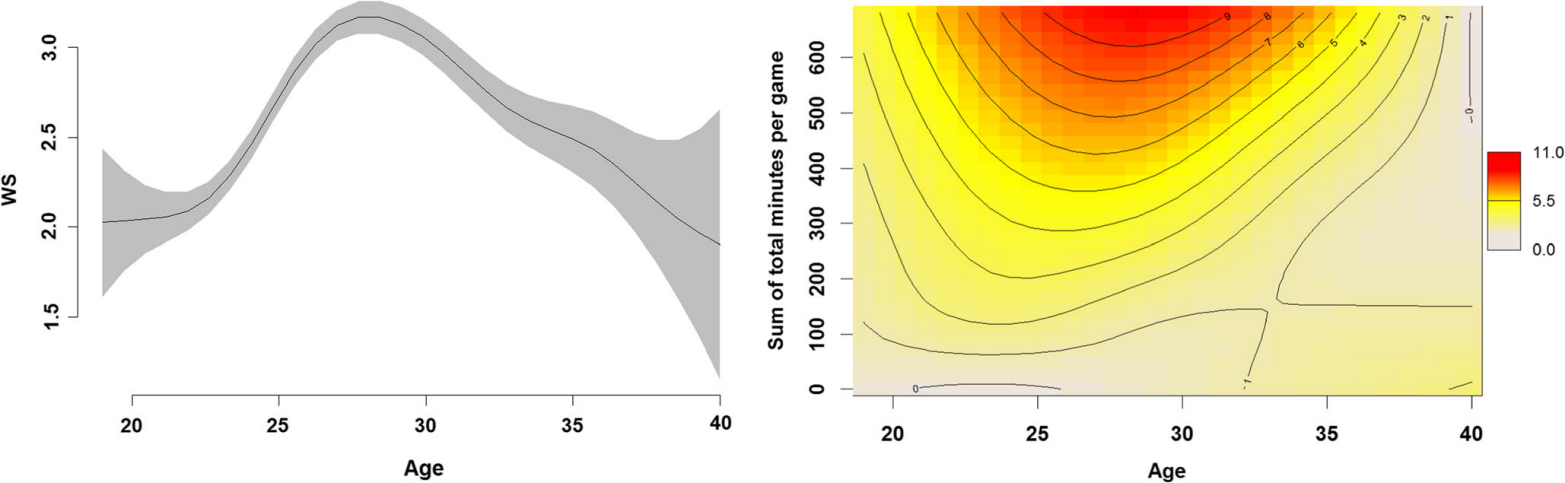
Illustration of the raw data for win share (WS) performance across players’ ages. The top panel illustrates nonlinear age-related changes in WS performance. The line indicates change in the mean WS performance across ages, whereas the shaded area shows 95% confidence intervals for the mean. The bottom panel illustrates the relation between the summed minutes per game individuals played during their lifetimes and their age-related changes in WS performance. The heat map indicates region of higher (red) and low (white) performance, as measured by WS and how this changes over age (*x*-axis) and total minutes per game played (*y*-axis)

In the first step of our investigation, we compared different exponential functions that can explain age-related changes in the case of basketball performance. An illustration of the raw data can be seen in Fig. 1, with general age-related changes (Fig. 7) and age-related changes moderated by players’ activity (total minutes per game played). To be able to estimate the complete form of the lifespan function, we separated the age-related changes into the prepeak increase and the postpeak decrease and modeled them as two different processes. For each of the two parts, we examined various exponential functions that can explain age-related changes (see Table 1). First, we included the power law function behind age-related increase and decrease in performance, as previous studies had shown that this function explains the majority of activity-related changes in performance (Newell & Rosenbloom, 1981; Ritter & Schooler, 2001). Second, we included an exponential growth curve as a potential underlying function, which had proved useful in previous studies (Schroots, 2012; Simonton, 1989, 1991, 1997). The third function that we examined was logistic growth, which is a good way of capturing the accumulation of knowledge (Keuleers et al., 2015). Finally, we included linear changes in the model, because these should indicate a potential constant increase to the peak and decrease after it (Roring & Charness, 2007; Salthouse, 2010, 2016).

**Table 1:** Mathematical functions used to model the age-related prepeak increase and postpeak decrease of performance in basketball

Function	Equation
Power law	$Performanc{e}_{pi}={\alpha}^{\ast } ag{e}_i^{\beta_p}$
Exponential growth	*Performance*_*pi*_ = *α*^∗^ exp(*β*_*p*_^∗^*age*_*i*_)
Logistic growth	$Performanc{e}_{pi}=\frac{\delta_p}{1+{\alpha}^{\ast}\exp \left({\beta_p}^{\ast } ag{e}_i\right)}$
Linear function	*Performance*_*pi*_ = *α* + *β*_*p*_^∗^*age*_*i*_

The parameters in different models refer to the same age-related process. *Performance* refers to the rating of an individual player (*p*) at a time point (*i*). The *α* parameter estimates the starting number of performance points, the *β* parameter estimates the rate of the change, and the *δ* parameter estimates the upper limit or maximal level of performance

The power law and logistic growth functions indicate that the age-related increase in performance slows as players reach the peak of their performance. In other words, the accumulation of skill is rapid at the beginning of the skill acquisition period, when every exercise brings new gains. As players get older, the increase in performance slows down and approaches the upper plateau asymptotically. The main difference between these two functions is in the beginning of the skill acquisition period, during which the logistic function predicts slower acquisition of the skill. In contrast with the logistic and power law functions, the exponential function does not assume that the speed of skill acquisition decreases at the peak, but rather that it continues in the same manner. Finally, the linear function assumes that the acquisition of skill follows a continuous increase until the peak level of performance. The same interpretation of exponential functions applies to the age-related decrease in the second part of the lifespan, when the age-related changes become more negative.

We used Bayesian hierarchical modeling, in which the exponential functions were fitted separately for the prepeak increase and postpeak decrease for every player in the dataset, as well as the age at which the increases in real-life performance transition to a consistent decrease. Following the lifespan theory, we do not assume that all the individual processes that underlie real-life performance decline at the estimated age, nor that all players decline at the same time. However, this is the moment when the product of individual processes results in negative changes. We employed an ad hoc division of the age-related function in order to test the shape of the functions, and model-based estimation of the inflection point to test the age at which this transition between two functions occurs. To estimate the age at which the transition between two functions occurs, we used step or Heaviside functions (Bracewell, 1986), which govern the conditional influence of the parameters in the model (see the Inflection Point section in the online materials). In the case of our model, the Heaviside function defines and estimates the age at which the prepeak increase changes to postpeak decrease. This type of model is often referred to as a *broken-stick* model (Flora, 2008; Hall, Ying, Kuo, & Lipton, 2003; Lange, Carlin, & Gelfand, 1992). The simple broken-stick model, which calculates the pre- and postpeak slopes as well as the inflection point, was estimated on the sample data (400 random players) with 50,000 samples as the adaptation phase, 10,000 burn-in samples, and 5,000 samples with a thin factor of 5.

To test the shape of the functions, we chose age 27 as the value at which the prepeak increase would turn toward the postpeak decrease. This value was based on estimates from previous studies (Benedict, 2017; Faulkner, Davis, Mendias, & Brooks, 2008; Lailvaux et al., 2014; Schulz et al., 1994; Wakim & Jin, 2014), reflecting that the aggregated performance across players becomes negative at 27 years, and the results from Heaviside functions showed that the inflection point falls within this age range. Although ad hoc division of the peak value can result in individual player biases to the overall functions, this approach decreased the complexity of the overall model, since it was not necessary to calculate the peak for every player in the dataset. For the shape of the functions, we ran 5,000 samples as an adaptation phase, 8,000 burn-in samples, and 5,000 samples with a thin factor of 5.

To investigate how well the mathematical functions fit the observed data, we used the deviance information criterion (DIC) as an analogue to the Akaike and Bayesian information criteria. All of these measures indicate the relative quality of a statistical model for a given dataset. DIC is particularly suited to Bayesian model estimation in which the results have been obtained by Markov chain Monte Carlo (MCMC) simulations. As well as measuring how well the model fits the data, the information criterion penalizes for the number of parameters used. When comparing two models with equal fits to the data, the more complex model will have a higher (i.e., worse) DIC measure than the less complex one.

### Age of inflection

To estimate the age of inflection, we calculated the global intercept and individual slopes for the age variable, and the age of inflection was then adjusted for every player in the data. Age was centered at 0, to make the estimate of the cutoff point easily interpretable, and we fitted models with linear and exponential functions (see the Inflection Point section in the online materials). The results showed that, with all three measures, the change in net performance occurred at similar ages: Players increase in performance up to 28 or 29 years old and start declining from that point on, whereas the individual inflection points ranged from 24 to 33 years for all three performance measures. In other words, the function that models the increase in performance is active until age 28 (including the age of 27) to 29 (including the age of 28), when the slope that governs the decrease becomes active. The results from our model confirmed findings from previous studies that have shown that the peak should be expected at around 27 or 28 years old.

### Shape of the function

The results show that in the case of a prepeak increase, exponential and power-law functions resulted in the lowest DIC, when compared with the other functions (see “Prepeak” in Table 2). In the case of WS, the prepeak increase follows an exponential function, reflecting that the rate of increase in players’ skill increases more rapidly than a linear change. The VORP initial increase follows a power-law function, whereas PER is best described by a linear increase (see Table 4 in the Appendix). For the postpeak decrease, the results show that the power-law function has the lowest DIC measure across all three measures of performance (see “Postpeak” in Tables 2 and 4). These results indicate that basketball players decrease in skill quickly after the peak of performance, but the rate of the decline is not constant and slows down in older age.

**Table 2:** Deviance information criteria for exponential functions used to model age-related changes in WS, given separately for the prepeak increase and postpeak decrease

	WS	
	Prepeak	Postpeak
Power law	51,118	34,974
Exponential	50,911	44,277
Logistic	56,360	NA
Linear	51,515	35,345

The NA (estimate not available) indicates that the models with the logistic function for the postpeak decrease could not be estimated

In addition to comparing the different functions that can explain age-related changes in performance, we also investigated the necessity of adjusting the random structure of the models. We compared models with and without random adjustments for the intercept and slope of the final function used. The results indicate that the chosen slope adjustment for each player in the dataset was well founded, whereas the intercept adjustment did not seem to be necessary. However, once we adjusted the slope for every player in the data, the general slope of the function lost statistical significance. Even though we are not primarily interested in *p* values and the interpretation of significance, this is a potentially interesting result that may indicate a high degree of variability in the increases and decreases in performance over a player’s career (see Table 3, as well as Table 5 in the Appendix). This suggests that some players, in fact, do not decline as they age, or that the additional increase in the first part of the career is limited to players who are already highly skilled on entering the league. Overall, the results indicate that age-related changes isolated from other potentially impactful variables do not have great explanatory power for the players’ performance in basketball.

**Table 3:** Estimates of the intercept and the slope for WS, given separately for the prepeak increase and postpeak decrease

		Function	Parameters	
			Intercept	Slope
			Mean (95% CI)	Mean (95% CI)
WS	Prepeak	Exponential	1.34 (1.27 to 1.40)	.000 (– .00091 to .0020)
	Postpeak	Power law	2.00 (1.96 to 2.16)	– .012 (– .022 to – .0018)

In the next step of the analysis, we combined the two functions that provided the best fit to the age-related changes, in a joint model that aimed to investigate how different variables influence these changes, and potentially to answer the question of increased variability in the changes. Finally, we calculated the correlation between the two phases, development and aging, to understand and produce a complete function of performance over players’ careers.

### Bayesian approach

The Bayesian statistical approach is based on the idea that probability can be defined as the degree of knowledge about a particular hypothesis (Gelman et al., 2014; Kruschke, 2014; Lee & Wagenmakers, 2014). The probability is expressed as the prior belief in or probability of an idea or hypothesis, which is updated when we observe the new evidence coming from the collected data, resulting in the posterior probability. In other words, the probability of a hypothesis is an orderly opinion expressed as a probability distribution, and inferences coming from the data (likelihood) offer revision of that opinion in light of relevant new information. In the Bayesian approach, we can easily use these probability distributions to represent knowledge and uncertainty about the variables of interest. More importantly, this knowledge can be processed, summarized, updated, and manipulated using the laws of probability theory (Lee, 2004, 2008).

One of the prominent ways in which the Bayesian approach can be employed is to build models that relate psychological processes to the observed data (Lee, 2004; Lee & Wagenmakers, 2014; Scheibehenne, Rieskamp, & Wagenmakers, 2013). This is not identical to the data analysis approach, in which practitioners use statistical tests, such as analysis of variance, to test a theoretical assumption. Instead, the goal is to create a more detailed statistical model of a particular aspect of cognitive functioning or behavior and to relate this model to the data. The memory retention or diffusion models are good examples of such an analysis, where the estimated parameters describe the decay rate of information and the drift rate (accumulation of information) over time (Ratcliff & McKoon, 2008). These parameters represent aspects of the assumed cognitive or theoretical process, which can then be isolated and investigated in more depth. This is one of the main reasons why we decided to use the Bayesian approach to data modeling. Leaving aside the oft-reported benefits of Bayesian analysis, we primarily used this environment because the proposed B-Ianus model requires high-dimensional integration with no known analytical solution. In other words, we do not have mathematical optimizers for the likelihood functions with which the B-Ianus model operates. This is mainly due to the necessity of investigating the interaction of lifelong changes with potentially interesting covariates. In this case, Bayesian modeling that relies on numerical integration methods such as MCMC (Robert & Casella, 1999) can estimate the parameters of the proposed model by sampling the values of the parameters from simulated posterior distributions.

### Bayesian latent cognitive variable modeling

In this study, we combined age-related modeling procedures with factor analysis to build a more informative model of age-related changes in real-life skills. In the first part of the modeling process, we used exponential functions to investigate age-related changes in performance in basketball (as we introduced in the Age-Related Changes section of the introduction). In the present section of the study, we show how the age-related functions can be interacted with variables of interest using latent variables, which results in a cognitive latent variable model (CLVM; Vandekerckhove, 2014). Therefore, we combined exponential modeling of age-related changes with individual differences in performance (Cronbach, 1957). The model we propose is built on three different levels: random effects, manifest predictors, and latent predictors.

**Fig. 2:**
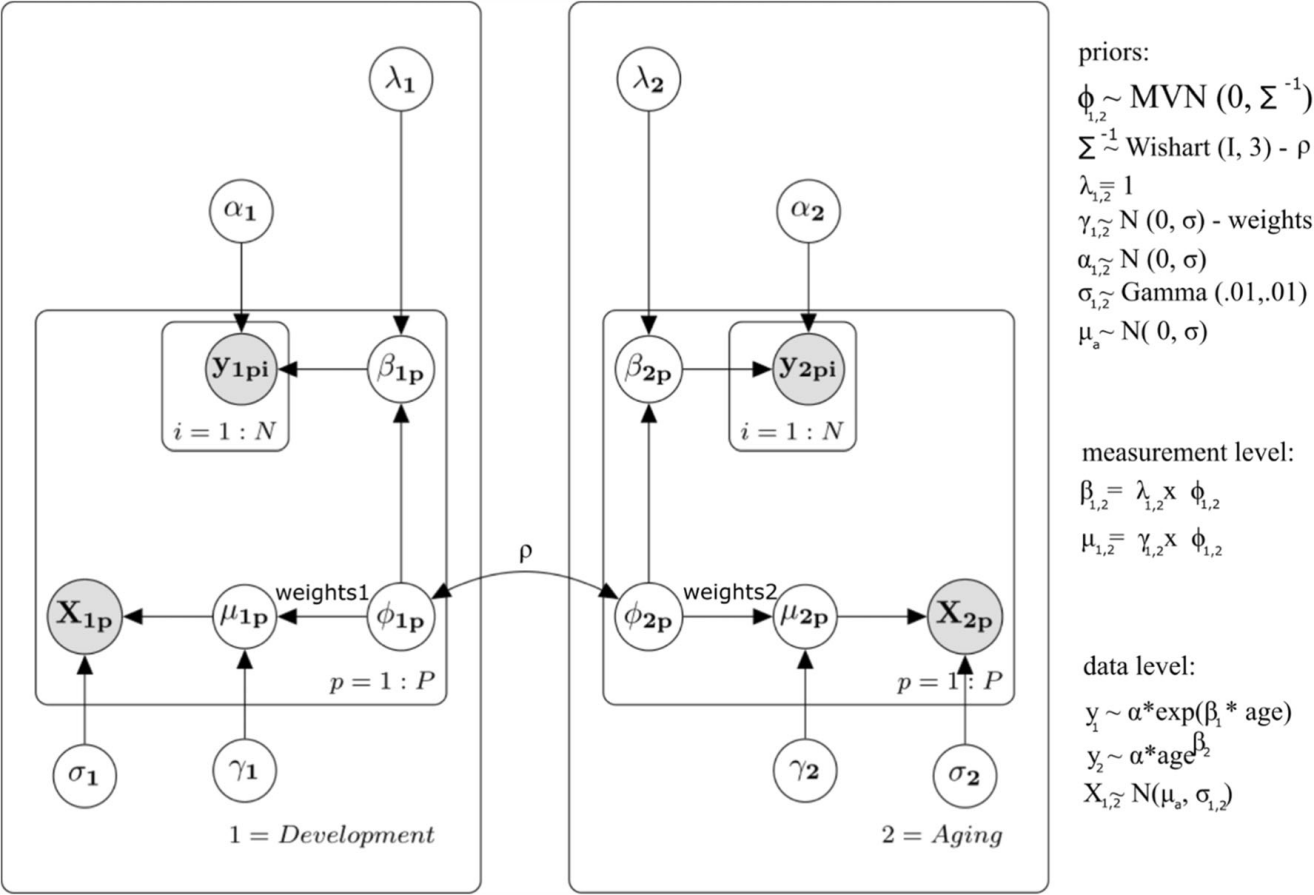
The graphic representation of the B-Ianus model of age-related changes in sports (Model 1). Plates 1 and 2 indicate prepeak increases and postpeak decreases in performance, which were modeled separately. In each plate, *p* shows independent repetitions over participants, and *i* shows independent repetitions over trials. The *α*_1_ and *β*_1_ parameters represent the increase in performance, whereas the *α*_2_ and *β*_2_ parameters model the decrease of the performance across age. The *β*_1_ and *β*_2_ parameters are loaded onto the latent development (*ϕ*_1_) and aging (*ϕ*_2_) factors by setting the *λ*_1_ and *λ*_2_ constraints. The covariates—minutes per game and position (*X*_1p_ and *X*_2p_), in our case—are loaded onto the respective latent factors through the *weight* (*γ*) parameters. Finally, the B-Ianus model also estimates the relation between the development factor and the aging factor, which is done by means of the *ρ* parameter

The first level of the model represents a random-effect structure for the parameters of interest. This is a set of parameters that are assumed to be drawn from some superordinate distribution (Baayen, Davidson, & Bates, 2008; Radanović & Vaci, 2013). For example, the sampled participants in the experimental setting represent just a small fraction of the variance of, for example, basketball skill in the whole population. In the case of this study, we adjusted the *β* parameter (slope) of the increase and decrease functions for every player in the database. By doing so, we modeled the rate of growth and decline of performance for every player over the course of their career. The individual slopes are defined as *β*_1p_ (prepeak) and *β*_2p_ (postpeak) in the Fig. 2.

The second level of the model comprised the manifest predictors—all the measures that can explain variability in the dependent variable. There are multiple ways in which researchers can model these predictors, from an analysis of variance or regression approach, in which we usually assume a linear structure between the dependent and independent variables, to nonlinear regression analysis (e.g., GAMs) and cognitive models (e.g., diffusion models). We used the previously explored exponential functions that capture age-related changes in the measures of performance (WS, VORP, and PER), but we also included the playing position of the player and how many minutes per game were played during a player’s prepeak and postpeak career periods. The manifest predictors are illustrated with the shaded nodes in Fig. 2.

The third level in the model comprised the latent factors, which offer us a joint explanation of the covariance between the set of observed variables. In other words, the latent factors are not observed but are estimated from the covariance matrix of the observed variables. Even though this approach is often used in the psychology of personality and intelligence, estimation and assumptions regarding potential latent structure are rarely used in age models (but see Oberauer, Süß, Schulze, Wilhelm, & Wittmann, 2000). In the case of our model, we included two latent factors that correspond to the skill acquisition and aging periods. Unlike previous models proposed in the domain of age-related changes, latent factors offer the possibility of modeling age-related changes in performance together with other possibly interesting variables (Goal 3). Latent factors are notated as the *ϕ* nodes in Fig. 2.

Before going into the results, we will explain the basis of the model step by step (for a detailed introduction to CLVM, see Vandekerckhove, 2014). For the data level, we used exponential functions that modeled the age-related changes, as well as the positions of the players and the total number of minutes played during the prepeak and postpeak periods. In the next step, we specified two latent factors of age-related changes, prepeak development and postpeak aging. In essence, we used the confirmatory model approach in a factorial analysis. Factorial analysis is usually represented by the linear equation *Y* = **Λ** * **Φ** + **E**, where **Φ** is the matrix of person-specific factor scores, **Λ** is a matrix of factor loadings, and **E** is a matrix of independent, zero-centered, normally distributed errors. Because the latent factors are not at a level accessible to measurement and are, therefore, completely theoretical, we needed to define which manifest variables are allowed to be related to the latent variable. The usual approach is to relate the first group of manifest variables to the first latent factor and the second group to the second latent factor, usually known as a *simple structure* or *congeneric factor* model (Anderson & Gerbing, 1988; Meredith, 1993).

Two different versions of the model were tested. The first version had the cutoff age for prepeak versus postpeak manually defined by the authors. In the second version, this inflection point was automatically calculated using Heaviside functions (see “B-Ianus Model 1” and “Model 2” in the Model section of the online materials). Besides the difference in defining the inflection point across ages, the two models differed slightly in overall structure. Model 1 had two separately defined likelihoods, one for the prepeak increase and one for postpeak decrease. Model 2 had only one defined likelihood, for age-related changes in performance. This difference between specification of the likelihoods results in different complexity, when it comes to the estimation and retrieval of parameters. Model 2, with automatic estimation of the inflection point, had higher complexity, since it had an additional parameter (the inflection point) and slope parameters that were jointly estimated. In combination with the latent structure, the model had a problem converging and retrieving the possible parameter values in a feasible number of MCMC runs. In contrast, Model 1, with separate likelihoods, converged on the highest probable value of the parameters in the same number of runs.

In the case of both models, the rate of increase was loaded onto the first factor, whereas the rate of decrease was loaded onto the second factor. The players’ position and total minutes played per game were loaded onto both factors. We aggregated these variables for every player into the prepeak and postpeak values, choosing 27 years as a cutoff value. Additionally, the loading values (*λ* nodes in Fig. 2) for the slope of the prepeak increase and postpeak decrease functions (*β* parameters) were fixed at a value of 1 for each factor: In this way, the measurement of a latent factor was defined as the rate of performance change before and after the peak. Consequently, this allowed us to investigate how the position played and the minutes contributed to each game correlated with the change in performance with age for basketball players. Positive *β* values in the developmental latent factors meant that players improved more dramatically, whereas positive *β* values in the aging factor meant that players decreased in skill at a slower rate. Finally, we specified the correlation structure between the two latent factors and investigated how the rate of prepeak increase influenced the postpeak decrease of performance in NBA players (lifespan interaction, illustrated as the *ρ* parameter in Fig. 2).

The complete overview of the model is illustrated in Fig. 2. Starting from the bottom of the graph, we can see that the change in performance is modeled as an exponential function for the prepeak increase, and a power-law function for the postpeak decrease. These functions were adjusted for every measure of performance—that is, for WS, VORP, and PER. The *β* (rate of change) was estimated for every player in the database. Therefore, the *β*s are drawn from superordinate distributions that tell us about individual differences in these parameters. On the second level, expertise-related activity, expressed as the total number of games played, together with the *β* parameter, is loaded onto the latent factors (skill acquisition and aging). The *λ*_1,2_ and *weight*_1,2_ parameters are the loadings, respectively, of the rate of change and the player’s position or total minutes per game on the latent factors, where the *λ* parameters are constrained to be 1. Therefore, the measurement scale of the latent factors is inferred from the slope of the age-related changes. This allows us to investigate all other auxiliary variables that could influence age-related changes in performance, such as intelligence, motivation, and personality dimensions (Bilalić, McLeod, & Gobet, 2007a, 2007b; de Bruin, Rikers, & Schmidt, 2007; Burgoyne et al., 2016; Charness, Tuffiash, Krampe, Reingold, & Vasyukova, 2005; Ericsson, Krampe, & Tesch-Römer, 1993). In other words, the model offers the possibility of investigating the building blocks of age-related changes in real-life performance. Finally, we investigated the interactions between the two latent factors, by including a correlational structure between prepeak increase and postpeak decrease. This parameter offers the possibility of estimating the relationship between the skill acquisition function and the aging function. The same model is illustrated more graphically in Fig. 7.

### Estimation of the model

The full model that included all relations and underlying latent factors was calculated only for the random sample of 400 players. We also transformed the relation between the performance measures and age to an approximate linear relation. This improved model optimization and decreased the time required to sample the posterior distribution of the parameters. In particular, in the case of the power-law relationship, we logarithmically transformed both the performance measure and age, whereas in the case of the exponential relationship, only the performance measure was transformed with the logarithmic transformation. The final estimates can easily be transformed back to the original values by using exponential transformations on the predictions of the model. To estimate the model, we used the Amazon AMI service, utilizing its cloud computing capabilities and reducing the time necessary for sampling out the complex posterior distribution calculated by this model. For each batch, we used 50,000 samples to adapt the model, 10,000 burn-in samples, and 10,000 samples with a thin factor of three steps.

## Results

### Goal 1—Basic form of the age-related function (continued)

**Fig. 3:**
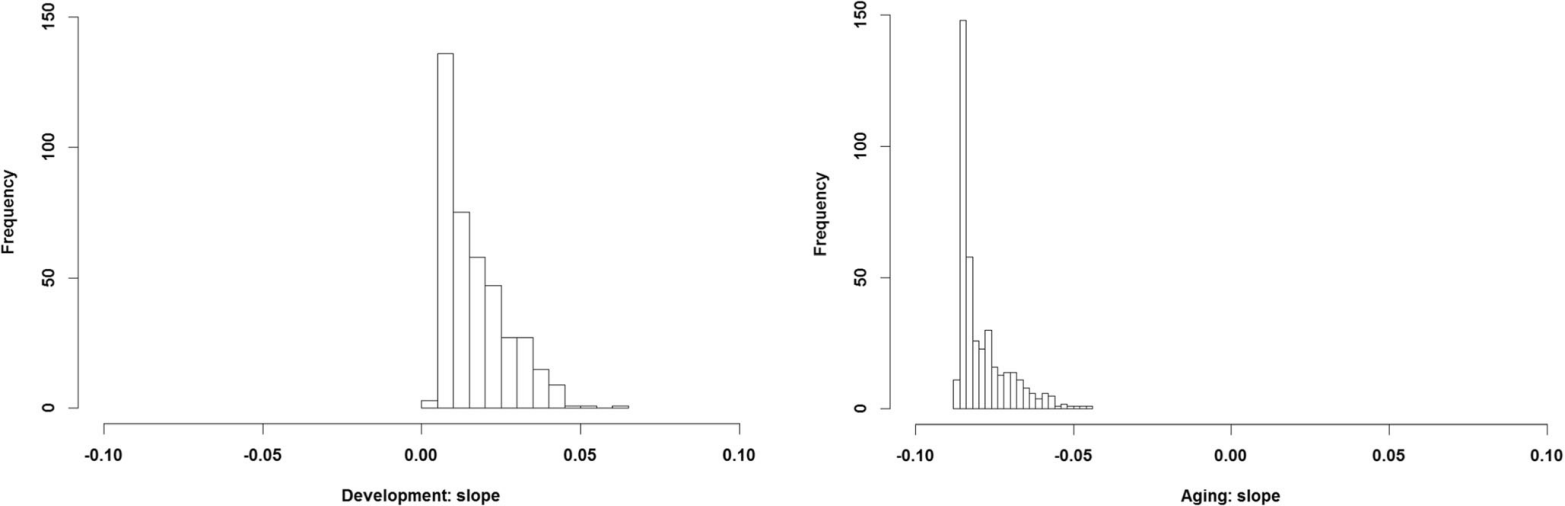
Estimated parameters for prepeak (development) and postpeak (aging) changes (slope of the function used). The panels show the possible values of parameters for the slope during the prepeak and postpeak periods when we approximated the exponential and power-law changes with linear functions. The intercepts of the functions were not adjusted for the individual players; thus, they were estimated to be equal, as in the case of the previous analysis (see the Shape of the Function section), and are not presented here

The intercept and the slope (the *α* and *β* parameters, respectively; see Fig. 3 for the slopes) were estimated similarly to those for the separate models for the development and aging functions (see the Goal 1 section above). The results indicate that people vary in the degree of change during the prepeak and postpeak, where a more positive parameter indicates either greater development or a more positive aging function—that is, less decline. The histogram plots show the possible values of parameters for the age-related changes and interactions. In the case of WS (see Fig. 3) and VORP (see Fig. 12 in the Appendix), the development slope is centered slightly above zero, with a prolonged tail on the positive values of the parameter. This shows that most players do improve during the first period of their career, whereas some players show a marked development, indicated by the large positive parameters. Negative values for the prepeak slope indicate that decline in performance can also occur during the prepeak phase. This is prominent in the case of the PER measure (see Fig. 8 in the Appendix), which seems to decline in the case of most players, indicated by the number of negative values.

In the case of the aging function, the results show that most players decline in all measures, since the highest density of the distribution covers negative values of the parameters.

### Interaction of prepeak and postpeak changes

**Fig. 4:**
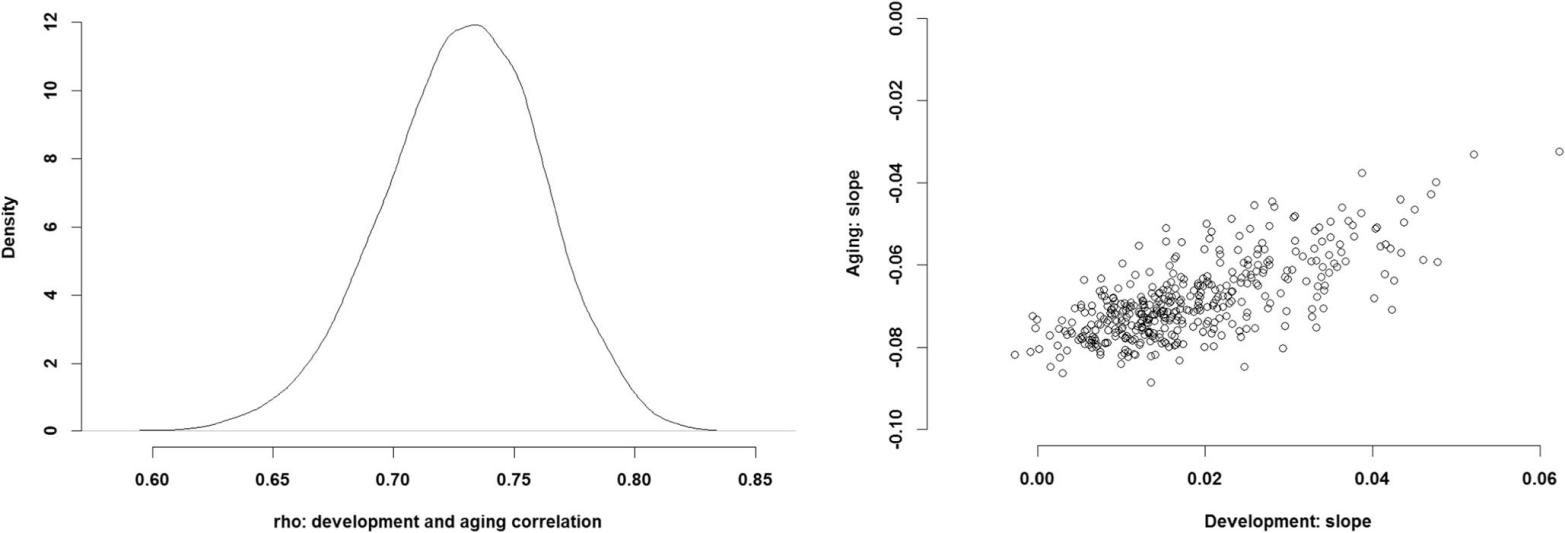
Interaction between the slope of the prepeak increase and the slope of the postpeak decrease for 400 random players in the database. (Left) Sizes of the correlation between the prepeak and postpeak slopes. (Right) Values for prepeak increase and postpeak decrease for the 400 random players in the dataset. The *x*-axis shows slope size for the prepeak change, whereas the *y*-axis illustrates slope size for the postpeak change. Positive values indicate a stronger increase and shallower decline, and negative or smaller values show a shallower increase to the peak and a stronger decline after it. The panels show possible values of the slope parameters for the prepeak and postpeak functions when we approximate the exponential or power-law relationship with linear functions. The slopes are estimated in log–linear and log–log space

As well as investigating the prepeak and postpeak changes, the B-Ianus model also estimates how these two periods interact with each other for each player in the dataset. In the case of all measures of performance, the results show that the rate of increase during the development phase correlates with the rate of decline in the aging phase (Fig. 4). In other words, the players that have a stronger and positive prepeak increase display a shallower and slower decline in later life, and the players with the strongest developmental phase barely decline in their performance at all. The additional measures also indicate similar interactions (see Figs. 9 and 13 in the Appendix)

### Goal 2: Group analysis

**Fig. 5:**
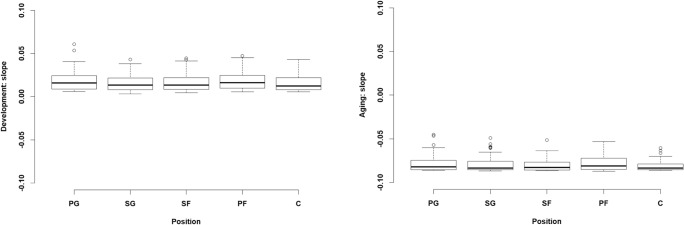
Differences in the size of the slopes for win shares, based on the different positions in basketball: PG, point guard; SG, shooting guard; SF, small forward; PF, power forward; and C, center. The *y*-axis illustrates the size of the slopes for the prepeak and postpeak changes, whereas the *x*-axis shows the different positions in basketball. The panels show the possible values of parameters for the slopes of the prepeak (left) and postpeak (right) functions when we approximated the exponential and power-law relationships with linear ones. The slopes in this case represent this relation in log–linear and log–log space

The B-Ianus model also offers the possibility of investigating whether overall age-related function differs between groups and individuals (the second goal of lifespan psychology). Here we included the position of the player as the numeric covariate in the model. The position of the player, together with the slope of the prepeak or the postpeak function, was regressed onto the latent factor. We used five main positions: PG, point guard; SG, shooting guard; SF, small forward; PF, power forward; and C, center. In this way, we investigated the potential interaction between the position of the players and the sizes of their slopes during age-related changes. The results show that player position does not change the slope of the increase or decrease during the prepeak and postpeak changes for WS, nor does it change it for other measures (see Figs. 5, 10, and 14 for the other measures).

### Goal 3: Building blocks of real-life performance

**Fig. 6:**
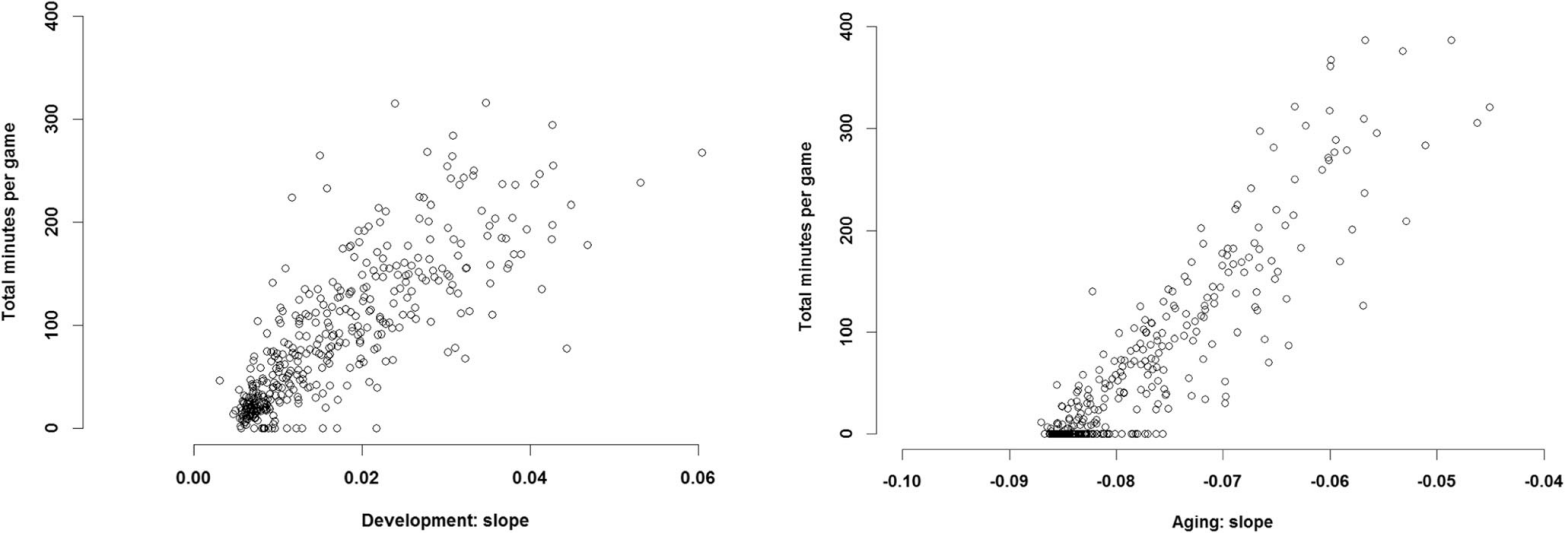
Interaction of prepeak and postpeak slopes for WS performance with the total minutes per game contributed by players during their careers. The *y*-axis illustrates the total number of minutes per game players contributed during the prepeak (left) and postpeak (right) periods, and the slopes of the functions, estimated from the prepeak and postpeak changes, are illustrated on the *x*-axis. Positive values of the slope indicate stronger positive change during the prepeak period and less decline during the postpeak period

**Fig. 7:**
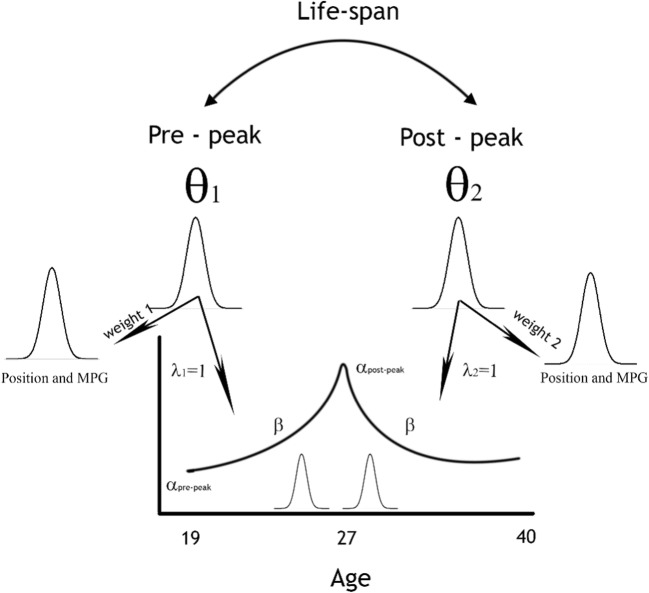
Illustrative representation of the B-Ianus model of age-related changes in sports (Model 1). Performance is modeled as a prepeak increase (before 27 years old) and postpeak decrease (after 27 years). The slopes of the increase and decrease (*β*) are adjusted for every participant, indicated by normal distributions as the basis of the functions. The slopes are loaded onto latent factors by the *λ* parameter; setting this parameter to 1 defines the measurement scale of the latent factor. The player’s position during his career and the total minutes per game were also loaded onto the latent structures through the *weight* parameters. Finally, the prepeak increase and postpeak decrease estimates, adjusted for other variables in the factorial structure, are correlated with each other. This is illustrated by the *Life-span* parameter

**Fig. 8:**
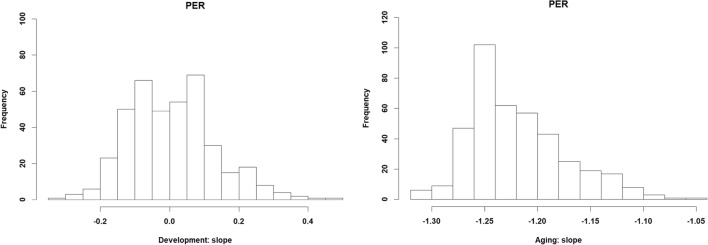
Estimated parameters for prepeak (development) and postpeak (aging) changes in PER. The panels show the possible values of parameters for the slope of the prepeak (left) and postpeak (right) functions when we modeled the PER changes with a linear function until the peak and approximated the power-law relationship with a linear one after the peak. The slopes in this case are represented in either linear (development) or log–log (aging) space

**Fig. 9:**
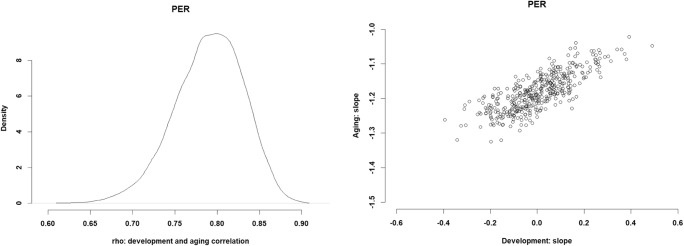
Interaction between the slope of the prepeak increase and the slope of the postpeak decrease in PER for 400 random players in the database. (Left) Sizes of the correlation between the prepeak and postpeak slopes. (Right) The *x*-axis shows slope size for the prepeak change, and the *y*-axis illustrates slope size for the postpeak change. Positive values indicate stronger increase and shallower decline, and negative values show a shallower increase to the peak and a stronger decline after it

**Fig. 10:**
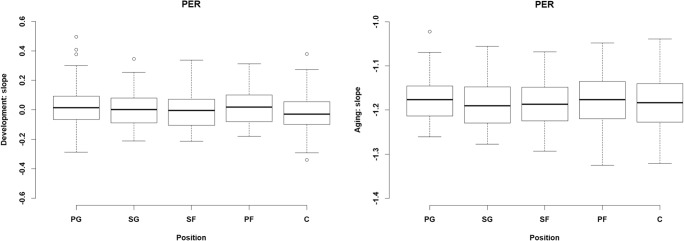
Differences in the size of the slope for the PER measure, based on the different positions in basketball: PG, point guard; SG, shooting guard; SF, small forward; PF, power forward; and C, center. The *y*-axis illustrates the sizes of the slope for the prepeak and postpeak changes, and the *x*-axis shows the different positions in basketball

**Fig. 11:**
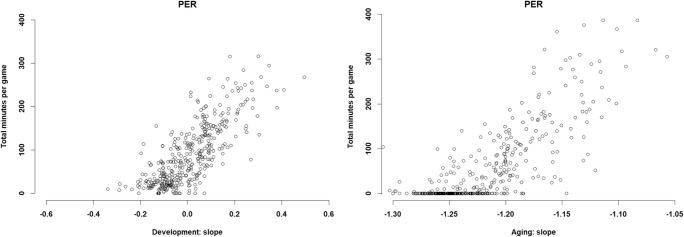
Interaction of the prepeak and postpeak slopes for PER performance with the total minutes per game contributed by players during their careers. The *y*-axis illustrates the total number of minutes per game players contributed during the prepeak and postpeak periods, whereas the slopes for the functions, estimated from the prepeak and postpeak changes, are illustrated on the *x*-axis

**Fig. 12:**
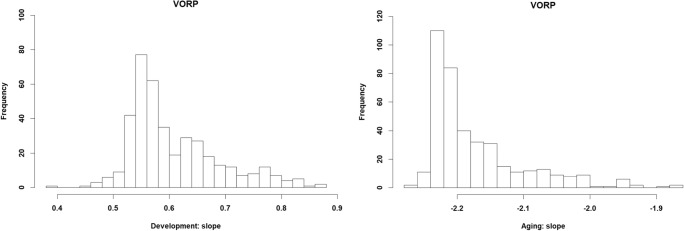
Estimated parameters for prepeak (development) and postpeak (aging) changes. The panels show the possible values of parameters for the slopes of the prepeak and postpeak functions when we approximated power-law changes in the VORP measure with linear functions before and after the peak. The slopes in this case are represented in log–log space (for development and aging)

**Fig. 13:**
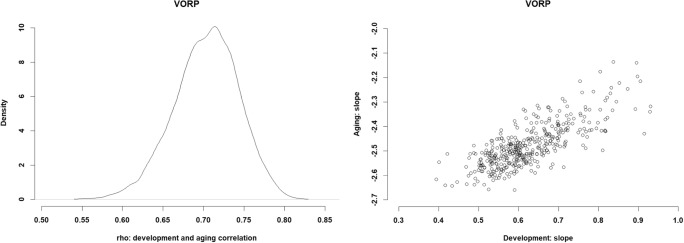
Interaction of the prepeak and postpeak slopes for VORP performance for 400 random players in the database. (Left) Sizes of the correlation between the prepeak and postpeak slopes. (Right) The *x*-axis shows slope size for the prepeak change, and the *y*-axis illustrates slope size for the postpeak change. Positive values indicate stronger increase and shallower decline, and negative values show a shallower increase to the peak and a stronger decline after it

**Fig. 14:**
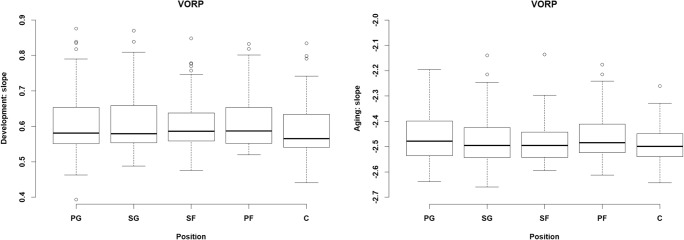
Differences in the size of the slope for the VORP measure, based on the different positions in basketball: PG, point guard; SG, shooting guard; SF, small forward; PF, power forward; and C, center. The *y*-axis illustrates the sizes of the slope for the prepeak and postpeak changes, and the *x*-axis shows the different positions in basketball

**Fig. 15:**
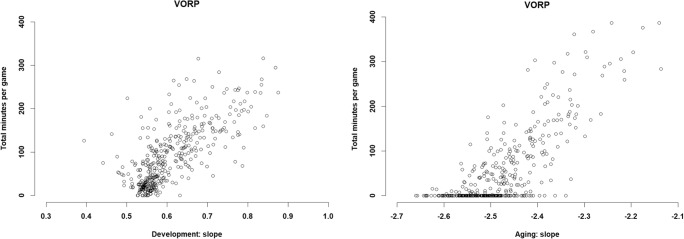
Interaction of the prepeak and postpeak slopes for VORP performance with the total minutes per game contributed by players during their careers. The *y*-axis illustrates the total number of minutes per game players contributed during the prepeak and postpeak periods, whereas the slopes for the functions, estimated from the prepeak and postpeak changes, are illustrated on the *x*-axis

In the final step, we investigated potential building blocks of the performance and different variables that can influence changes in performance (the third goal of lifespan psychology). We used the total minutes per game that players contributed during the prepeak and postpeak changes (for a similar measure, the total number of games played during the career, see the supplementary materials). The results show that, during the prepeak increase, contributing more minutes per game has a positive interaction with the slope. That is, the players who increase in performance during the first part of their careers also play more minutes per game. Similarly, players that play more minutes per game tend to decrease less in their performance than do the people contributing fewer minutes per game (see Fig. 6, as well as Figs. 11 and 15 for the other measures of performance).

## Discussion and conclusion

The relationship between performance and cognitive functioning, on the one hand, and age, on the other, has a long history of investigation. These changes have been investigated from different perspectives, ranging from skill acquisition studies, which focus on age-related improvements that take place in early life (Donner & Hardy, 2015; Gaschler, Progscha, Smallbone, Ram, & Bilalić, 2014; Heathcote, Brown, & Mewhort, 2000; Tenison & Anderson, 2016), to aging studies, which focus on decline in cognitive performance that occurs in later life (Bugg, Zook, DeLosh, Davalos, & Davis, 2006; Lindenberger & Ghisletta, 2009; Salthouse, 2001, 2010). Even though studies in these domains have provided an invaluable body of evidence on age-related changes, understanding of the general age-related function of real-life performance, as well as the factors that change this function, has proved elusive. We believe that this is due to two main reasons: the difficulty of obtaining large sample sizes of measurements in real-life domains and the complexity of modeling nonlinear changes in the aging function.

In this study, we have provided a potential solution to those problems, by proposing a methodological and statistical environment in which practitioners can investigate age-related changes in greater detail by modeling natural datasets of real-life performance in sports. We used Bayesian cognitive latent variable modeling (Vandekerckhove, 2014) to investigate age-related changes across the complete lifespan and to quantify the interactions between age-related improvements and declines in basketball performance. Besides investigating complete career performance, we also demonstrated how the age-related changes that occur over the course of a basketball player’s career can be jointly investigated with other variables of interest, such as the player’s playing position and activity levels.

In comparison with previously proposed models, such as the Janus model or the model of career trajectories and landmarks (Schroots, 2012; Simonton, 1991, 1997), the B-Ianus model offers estimation of additional parameters that are of interest for the investigation of different theoretical proposals. First, researchers can investigate the optimal underlying function that explains age-related changes in performance, and can use these functions to model the changes. In other words, the B-Ianus model allows practitioners to use different age-related functions (e.g., power law, exponential, linear), whereas previous models have used prespecified double-exponential or double-logistic curves. Second, it is possible to adjust the random structure of the model: That is, we can estimate individual differences in the parameters that are used to describe age-related changes. In this way, practitioners can calculate individual peaks (maxima) or the rate of performance change for individual people or different groups of individuals. Adjustment of the random structure and individual differences are particularly useful when dealing with complex data, since this flexibility solves problems with the aggregation of the data (Heathcote et al., 2000).

Another advantage is that the random adjustments help alleviate the problems of outliers, since these values shrink toward the mean of the distribution for a particular parameter (Baayen et al., 2008; Radanović & Vaci, 2013). The model also allows for the inclusion of time-invariant variables and investigation of their relationship with age-related changes over the course of the career. Ultimately, B-Ianus enables us also to investigate interaction between the rates of prepeak increase and postpeak decrease. The model estimates the potential relationship between the growth rate in the first function and the rate of decrease in the second, given any other variable that can influence and change this relationship. The quantification of this parameter has proved elusive in the case of most modeling endeavors. In the case of polynomials, the individual parameters are (by the mathematical definition of a polynomial) correlated, even though this need not be the case with the data. Contrary to this, exponential functions require two-step procedures, where in the first step researchers estimate the prepeak and postpeak functions, and in the second step they calculate the correlation between these estimates. Finally, nonlinear models, such as GAMs, even though they calculate the optimal nonlinear function, provide this information only on the visual level, through interpretation of the fit of the model (illustrated in Fig. 1b). In practical terms, the B-Ianus model quantifies the relationship presented in Fig. 1b, in the case where we divide the age-related progression into prepeak increase and postpeak decrease.

Using the B-Ianus model, we showed how the three goals of lifespan psychology can be investigated in more detail. In the case of the basic form of the age-related function (Goal 1), we demonstrated that three frequently used measurements of basketball performance (WS, PER, and VORP) differ during the first part of the career, when which some of them change exponentially, linearly, or in a power-law fashion. It is likely that the different developmental patterns of these measures reflect differing cognitive processes (e.g., Heathcote et al., 2000; Newell & Rosenbloom, 1981), especially when we take into account the fact that the measurements aim to quantify different aspects of performance. Contrary to the prepeak development, the decrease after the peak, for all three measurements, follows a power-law function. People decrease rapidly immediately after reaching their peak, but as they age, this decrease in performance lessens and slows down. The B-Ianus model also indicated that there are no differences in the changes in performance over the career between the different positions that players play. The rates of increase and decrease—the slopes of the functions—do not change on the basis of playing position. In this way, we investigated the second goal of lifespan psychology—changes in the aging function—on the basis of subgroup analysis.

However, adding these types of covariates can also tell us more about the aging function, especially if we can identify people who drop out at an earlier or later stage of their career. Including this information in the model could potentially give more information regarding whether the decrease in the postpeak decline is influenced by dropout rates, where stabilization of the decline might occur just because of the higher-performing individuals. In addition, the B-Ianus model also showed that the number of minutes played per game correlates with the slopes of the prepeak and postpeak functions, thereby meeting the goal of lifespan psychology—that is, revealing the potential building blocks of real-life performance. The players who show greater development in the first part of their careers seem to contribute more minutes per game than their slower-developing teammates. The same result was obtained in the second part of the career, in that players who contribute more minutes per game display a shallower age-related function. In this particular instance, it is not possible to claim with certainty that the activity improves the skill acquisition process or reduces the negative age-related changes. The amount of time spent on court is dependent on how well players perform. Obviously, those who perform better at later stages of their career will play more than their less well-performing peers.

One of the most intriguing results in the present study is the B-Ianus parameter that estimates the interplay between prepeak and postpeak changes. The results on all three measurements of performance showed that players showing greater development during the first part of their careers displayed shallower declines in performance as they aged. This indicates potentially preserving effects of the skill acquisition phase, in which players who excel in the domain collect more knowledge and skill. On the one hand, basketball is a speed- and strength-dependent sport (Latin, Berg, & Beachle, 1994), in which greater declines in performance are expected (Faulkner et al., 2007; Goodpaster et al., 2006; Rogers & Evans, 1993; Thelen, 2003), given the general finding of more decline in physical than in cognitive domains (Fair, 2007). On the other hand, we were investigating a complex real-life skill for which people acquire a vast amount of knowledge and continue to do so in the later stages of their careers (as, e.g., in the case of vocabulary). In basketball, that knowledge is of a kinetic nature and may involve one’s own and one’s opponents’ movements, as well as team-specific patterns (Bilalić, 2017). Once the decline in performance begins, related to diminishing physical abilities, more knowledgeable or more able players may utilize their knowledge to preserve their current performance in the face of aging. It is possible that other factors—such as physical ability, personality, motivation, or even general genetic makeup—that enable certain players to acquire knowledge and skill more quickly may act as mediators of this correlation. In any case, the result runs counter to a large body of evidence that has demonstrated that age is not kinder to more able people (Blum & Jarvik, 1974; Vaci et al., 2015).

The B-Ianus model and the way it is estimated can be used in different domains of real-life performance, but also in domains in which researchers need to model nonlinear changes over time. In the case of this study, we showed how researchers can use it on a natural dataset collected in the basketball domain. The flexible nature of the Bayesian framework that underlies B-Ianus allows for the application of this model to any other domain.

### Author note

We are grateful to Rob Goldstone, Set Frey, and Tom Stafford for their comments on a previous draft of the article. Matthew Bladen’s assistance with preparing the text for publication is greatly appreciated.

## Appendix

### Other basketball performance measures (PER and VORP)

**Table 4:** Deviance information criteria for the functions used to model the age-related changes in VORP and PER, treating the prepeak increase and postpeak decrease separately

	PER		VORP	Postpeak
	Prepeak	Postpeak	Prepeak	
Power law	45,614	27,569	24,870	19,026
Exponential	45,307	28,176	25,118	20,000
Logistic	45,286	NA	NA	NA
Linear	45,032	27,580	25,252	19,264

NA (estimates not available) indicates that models with the specified functions could not be estimated for the prepeak increase or postpeak decrease.

**Table 5:** Estimates of the intercept and the slope for the PER and VORP measures, given separately for the prepeak increase and postpeak decrease

		Function	Parameters	
			Intercept	Slope
			Mean (95% CI)	Mean (95% CI)
PER	Prepeak	Linear	12.34 (12.01 to 12.51)	.0045 (– .0018 to .011)
	Postpeak	Power law	12.02 (11.81 to 12.28)	– .0029 (– .0072 to .0012)
VORP	Prepeak	Power law	0.062 (0.048 to 0.77)	.035 (.004 to .065)
	Postpeak	Power law	0.340 (0.304 to 0.374)	– .030 (– .048 to – .0115)

## Footnotes

1 The analyses presented here are a primer on how the B-Ianus model can be used. Analyzing all data would require computational resources that the authors currently do not have at their disposal. To calculate the model on the 400 players, we utilized Amazon AMI services that run a 3.0-Ghz Intel Xeon scalable processor with 32 GB of memory. Using this service, the estimation of the parameters was running for approximately 12 h per model. The high computational cost is the main limitation of the proposed model.
